# Entomopathogenic fungi as microbial platforms for tyrosine kinase-oriented inhibitor discovery

**DOI:** 10.1007/s00253-026-13975-y

**Published:** 2026-08-03

**Authors:** Victor Uchenna Chigozie, Kennedy Izuchukwu Ogunwa, Nkeiruka Grace Osuafor, Leslie Luxe Nduwimana, Daniel Ikechukwu Oraekei

**Affiliations:** 1 Department of Pharmaceutical Microbiology and Biotechnology, David Umahi Federal University of Health Sciences, Ebonyi Uburu, Nigeria; 2 NaPBiTReG (Natural Products Bioprospecting and Translation Research Group), International Institute for Pharmaceutical Research and Innovation (IIPRI), Uburu, Ebonyi Nigeria; 3 Department of Industrial and Medicinal Chemistry, David Umahi Federal University of Health Sciences, Uburu, Nigeria; 4 International Institute for Toxicology, Environmental & Occupational Health and Safety Research, DUFUHS, Ebonyi, Nigeria; 5 Department of Clinical Pharmacy and Pharmacy Administration, University of Abuja, FCT, Abuja, Nigeria; 6 Department of Pharmaceutical Microbiology and Biotechnology, Olivia University, Mukaza, Bujumbura Burundi; 7 Department of Pharmacology and Toxicology, Nnamdi Azikiwe University, Awka, Anambra Nigeria

**Keywords:** Entomopathogenic fungi, Tyrosine kinase inhibitors, Secondary metabolism, Biosynthetic gene clusters, Metabolic engineering, Natural product drug discover

## Abstract

**Abstract:**

Tyrosine kinase inhibitors (TKIs) remain a major focus of targeted therapeutic discovery, yet current discovery pipelines are constrained by resistance development, limited scaffold diversity, and high attrition rates. Entomopathogenic fungi (EPF) represent an underexplored microbial resource with significant biosynthetic potential for generating kinase-relevant secondary metabolites. Recent genomic studies reveal that EPF encode diverse and largely cryptic biosynthetic gene clusters (BGCs), including polyketide synthase (PKS), non-ribosomal peptide synthetase (NRPS), and hybrid pathways capable of producing structurally complex bioactive compounds. This mini-review examines the biotechnological exploitation of EPF for TKI discovery, focusing on fungal metabolites with direct or indirect kinase-related activity, genome mining strategies, biosynthetic logic, pathway regulation, and engineering approaches for metabolite production. Although only a limited number of EPF-derived metabolites have been experimentally validated as direct tyrosine kinase inhibitors, accumulating evidence indicates that many modulate kinase-associated signalling pathways and represent promising starting points for kinase-oriented natural product discovery. Particular emphasis is placed on pathway activation, heterologous expression, metabolic engineering, and scalable bioprocess development. Key translational challenges, including selectivity, toxicity, yield instability, and regulatory considerations, are also discussed. Collectively, this review highlights EPF as promising microbial platforms for kinase-oriented natural product discovery and provides a biotechnology-focused framework for advancing fungal secondary metabolites toward translational development.

**Key points:**

• *Entomopathogenic fungi encode diverse kinase-active secondary metabolites.*

• *Genome mining and synthetic biology unlock cryptic TKI pathways.*

• *Biotechnological optimization is critical for scalable fungal TKI discovery.*

## Introduction

Tyrosine kinases regulate cellular signaling pathways involved in proliferation, differentiation, metabolism, migration, and survival. Dysregulation of tyrosine kinase–dependent signaling contributes significantly to cancer, inflammatory disorders, and immune dysfunctions (Yagin et al. 2025). Consequently, tyrosine kinase inhibitors (TKIs) have become important targeted therapeutics in oncology and precision medicine. However, current TKI discovery pipelines remain constrained by resistance development, limited scaffold diversity, off-target toxicity, and repeated identification of chemically similar pharmacophores from synthetic libraries (Baldi et al. 2025). These limitations continue to stimulate interest in alternative discovery platforms capable of generating structurally diverse kinase-targeting molecules.

Despite intensive synthetic medicinal chemistry efforts, just few fundamentally new kinase-binding scaffold classes have entered development in recent years. This limitation has renewed interest in natural products, whose greater structural complexity and stereochemical diversity may provide access to previously unexplored regions of kinase-relevant chemical space.

Microorganisms remain major sources of pharmacologically relevant natural products, particularly structurally complex bioactive scaffolds (Chigozie and Esimone 2026a). Filamentous fungi are especially notable because of their dozens of secondary metabolic capacity, producing polyketides, non-ribosomal peptides, terpenoids, alkaloids, and hybrid metabolites with diverse biological activities (Riedling and Rokas 2025; Zhgun 2023). Genomic studies further reveal that many fungal biosynthetic gene clusters (BGCs) remain transcriptionally silent under standard laboratory conditions, indicating substantial unexplored metabolic diversity (Peni Wulandari et al. 2026). These findings have renewed interest in fungi as engineerable microbial platforms for biotechnology-driven natural product discovery (Chigozie and Esimone 2026b).

Among filamentous fungi, entomopathogenic fungi (EPF) including *Beauveria*, *Metarhizium*, *Cordyceps*, and *Isaria* represent promising but underexplored systems. Their specialized interactions with insect hosts require coordinated production of enzymes, toxins, and signaling-active metabolites capable of modulating eukaryotic cellular processes. Several EPF-derived metabolites modulate MAPK, PI3K/Akt, mTOR, and receptor-associated kinase pathways (Sheng et al. 2026; Morgos et al. 2024), supporting their relevance in kinase-oriented discovery. Recent advances in genome mining, metabolomics, synthetic biology, and pathway engineering have substantially improved access to fungal secondary metabolism. Platforms including antiSMASH, PRISM, and AI-assisted prediction tools facilitate systematic identification of cryptic BGCs (Chigozie et al. 2025; Hannigan et al. 2019). In parallel, CRISPR-based genome editing, promoter refactoring, heterologous expression, and pathway activation strategies are transforming fungi into programmable microbial chassis for biotechnology applications.

Rather than presenting EPF as established sources of validated tyrosine kinase inhibitors, this review evaluates their emerging potential as biotechnology-enabled platforms for discovering, engineering, and producing kinase-relevant secondary metabolites that warrant systematic kinase-focused investigation.

## Methodological approach

Literature included in this mini-review was identified through a structured narrative search focused on entomopathogenic fungi (EPF), fungal secondary metabolism, tyrosine kinase inhibitor (TKI) discovery, and microbial biotechnology. Searches were conducted using PubMed, Scopus, Web of Science, and Google Scholar, with additional studies identified through citation tracking of relevant articles and review papers.

Search terms included combinations of “entomopathogenic fungi,” “Beauveria,” “Metarhizium,” “Cordyceps,” “secondary metabolites,” “tyrosine kinase inhibitors,” “kinase signaling,” “biosynthetic gene clusters,” “fungal biotechnology,” “metabolic engineering,” “heterologous expression,” “genome mining,” “CRISPR,” and “natural products.” Emphasis was placed on peer-reviewed studies published primarily between 2010 and 2026, while seminal earlier reports were included where relevant.

Studies were prioritized based on relevance to: (i) experimentally validated kinase-related activity of fungal metabolites; (ii) biosynthetic gene cluster characterization; (iii) genome mining and metabolomics approaches; (iv) synthetic biology and metabolic engineering applications; and (v) fermentation and bioprocess optimization strategies. Particular attention was given to studies employing biochemical kinase assays, multi-omics analyses, heterologous expression systems, and molecular approaches supporting mechanistic interpretation. Because relatively few studies directly investigate tyrosine kinase inhibition in EPF-derived metabolites, evidence from related filamentous fungi was selectively incorporated where it provided mechanistic, biosynthetic, or biotechnological insight relevant to EPF systems. Reports based solely on preliminary phenotypic observations without mechanistic support were interpreted cautiously.

This review therefore aims to synthesize current knowledge regarding the biotechnological potential of EPF as platforms for kinase-oriented natural product discovery, biosynthetic engineering, and translational development. Given the limited availability of direct biochemical evidence, conclusions throughout this review distinguish validated tyrosine kinase inhibition from indirect modulation of kinase-associated signaling pathways to provide a balanced assessment of the current evidence.

## EPF as reservoirs of kinase-active metabolites

**Fig. 1:**
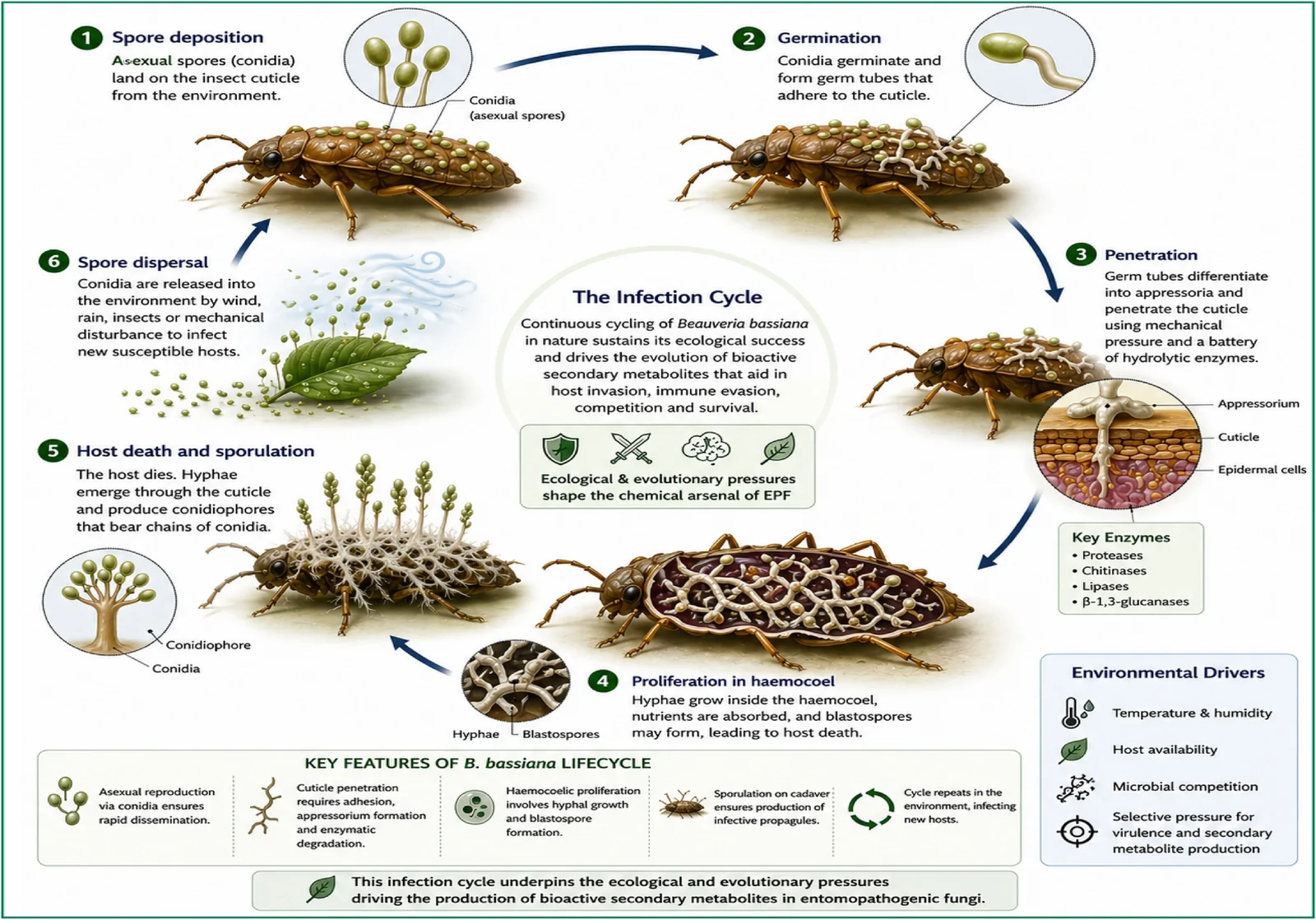
Lifecycle of *Beauveria bassiana* illustrating host infection and propagation. Lifecycle of *Beauveria bassiana* illustrating host infection and propagation, from conidial attachment and cuticle penetration to haemocoel colonization, host death, and external sporulation, highlighting the ecological pressures that drive the evolution of bioactive secondary metabolites in entomopathogenic fungi. (Authors concept image)

Entomopathogenic fungi (EPF) are increasingly recognized as metabolically versatile microorganisms with substantial potential for biotechnology-driven natural product discovery. Traditionally investigated for insecticidal activity and biological control applications, EPF also synthesize structurally diverse secondary metabolites with activities extending beyond insect pathogenesis (Pedrini 2022; Mantzoukas et al. 2022). Advances in fungal genomics, metabolomics, and biosynthetic pathway analysis have revealed that EPF harbor a good number of biosynthetic gene clusters (BGCs) encoding chemically complex metabolites with potential relevance to kinase-associated signaling pathways (Zhgun 2023). Major EPF genera including *Beauveria*, *Metarhizium*, *Cordyceps*, and *Isaria* occupy specialized ecological niches that impose strong selective pressures on metabolic adaptation. Successful host infection requires coordinated production of hydrolytic enzymes, immune-modulating compounds, cytotoxic metabolites, and signaling-active molecules that facilitate proliferation within the insect haemocoel (Zhang et al. 2025a, b). The infection lifecycle and associated ecological pressures underlying secondary metabolite evolution in EPF are summarized in Fig. 1. These prolonged interactions with eukaryotic hosts may contribute to emergence of metabolites capable of modulating conserved phosphorylation-dependent signaling pathways. Compared with many saprophytic fungi, EPF experience sustained evolutionary pressure to interact with living eukaryotic hosts, potentially enriching their metabolomes in signaling-active compounds. Although this hypothesis requires further experimental validation, it provides a biologically plausible rationale for prioritizing EPF within kinase-oriented natural product discovery programs. EPF produce diverse metabolite classes including polyketides, non-ribosomal peptides, cyclodepsipeptides, diketopiperazines, terpenoids, alkaloids, and hybrid PKS–NRPS compounds. Representative metabolites such as beauvericin, bassianolide, destruxins, oosporein, tenellin, and cordycepin modulate MAPK, PI3K/Akt, NF-κB, mTOR, and receptor-associated signaling pathways in mammalian systems (Sheng et al. 2026; Morgos et al. 2024; Toopaang et al. 2023; Qin et al. 2019). Although direct biochemical validation of tyrosine kinase inhibition remains limited for many EPF metabolites, these pathway-level effects support their relevance within kinase-oriented discovery pipelines. These observations should not be interpreted as evidence that these metabolites are validated tyrosine kinase inhibitors but rather as justification for their prioritization in future kinase-focused biochemical screening programmes.

Current understanding of EPF metabolic diversity remains incomplete. Whole-genome sequencing consistently reveals substantially more predicted BGCs than experimentally characterized metabolites, indicating extensive cryptic biosynthetic capacity (Meesil et al. 2025). In *Beauveria bassiana* and *Metarhizium robertsii*, a number of PKS, NRPS, terpene synthase, and hybrid clusters remain transcriptionally silent under standard laboratory conditions (Toopaang et al. 2025). Emerging approaches including genome mining, epigenetic modulation, co-culture systems, stress induction, and synthetic biology-based pathway activation are increasingly used to access these silent metabolic reservoirs. Importantly, the disparity between predicted biosynthetic capacity and experimentally characterized metabolites suggests that current limitations reflect incomplete pathway activation and metabolite characterization rather than exhaustion of EPF chemical diversity. Consequently, the principal discovery bottleneck has shifted from genome sequencing to functional validation of cryptic biosynthetic pathways.

EPF possess several features supporting their suitability for kinase-active metabolite discovery. Their metabolites exhibit structural complexity and stereochemical diversity difficult to reproduce synthetically (Zhang et al. 2026; Bihal et al. 2023; Chigozie and Esimone 2026b), while modular biosynthetic pathways permit pathway engineering, promoter replacement, domain swapping, and heterologous expression for metabolite optimization and analogue generation (Meng et al. 2022). Advances in metabolomics-guided screening and kinase-focused bioassays further support systematic interrogation of EPF metabolite libraries (Zhang et al. 2026).

Nevertheless, caution is required when interpreting pathway modulation data because many studies rely primarily on cellular phenotypes or downstream signaling markers rather than direct biochemical kinase assays. Distinguishing direct target engagement from indirect pathway perturbation therefore remains essential for translational development of credible TKI lead compounds. Current evidence supports EPF as underexplored microbial reservoirs of chemically diverse and biologically relevant scaffolds with potential applications in kinase-oriented therapeutic discovery.

## Evidence for tyrosine kinase inhibitor activity in fungal metabolites

**Fig. 2:**
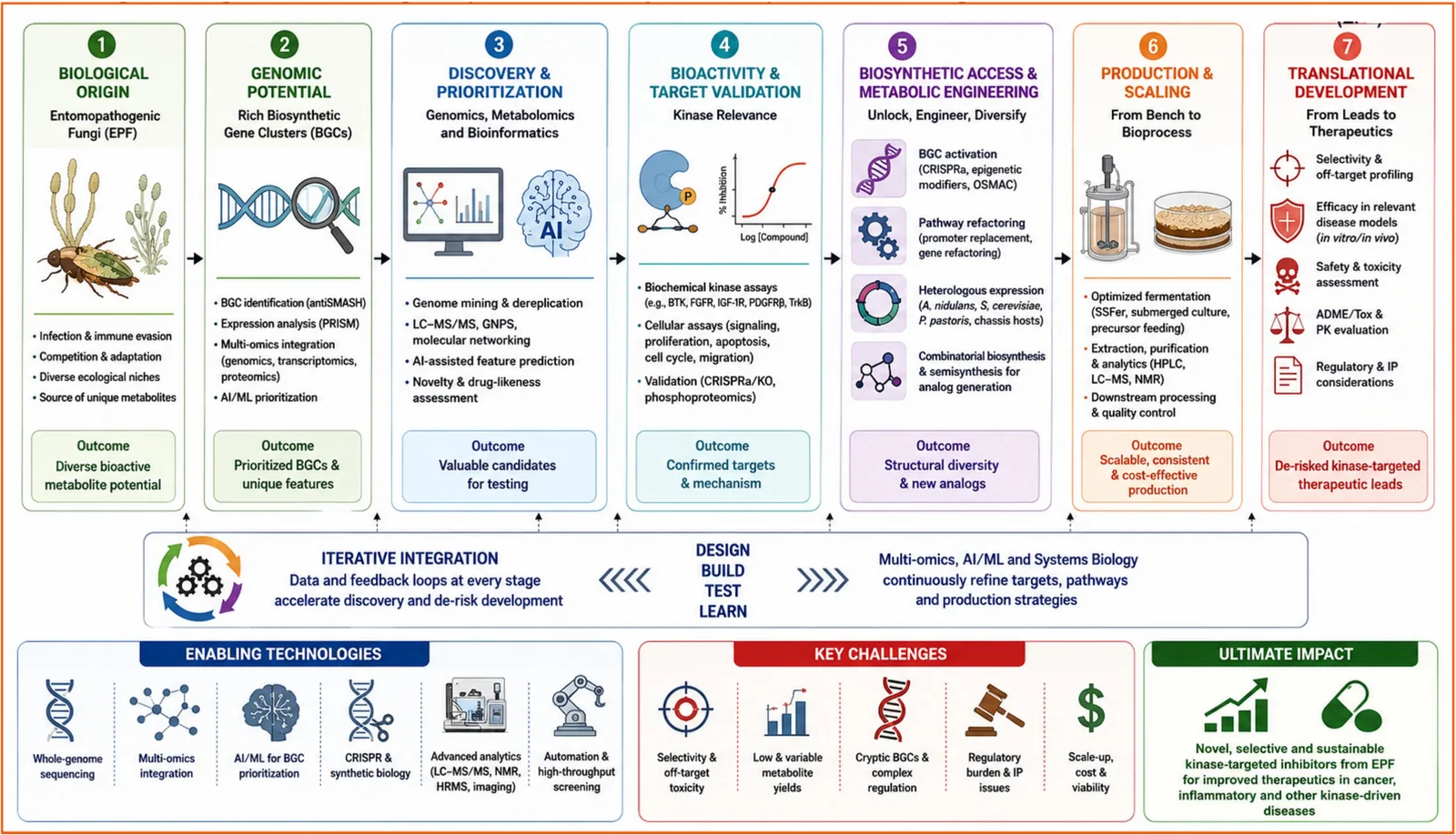
Biotechnological exploitation of entomopathogenic fungi for kinase-oriented natural product discovery (genomic potential to translational impact)**.** Integrated biotechnology pipeline for EPF-derived tyrosine kinase inhibitor discovery, linking genome mining and multi-omics validation to synthetic biology–driven pathway engineering, scalable bioprocessing, and lead optimization within an iterative design–build–test–learn framework (*authors concept image)*

Fungal secondary metabolites have contributed important pharmacologically active scaffolds to drug discovery, yet their potential as sources of tyrosine kinase inhibitors (TKIs) remains comparatively underexplored. Increasing evidence indicates that metabolites derived from entomopathogenic fungi (EPF) and related filamentous fungi can directly inhibit tyrosine kinases or modulate kinase-dependent signaling pathways associated with proliferation, apoptosis, inflammation, and cellular stress responses (Abulaiti et al. 2024; Zhang et al. 2020). These findings support the feasibility of exploiting fungal biosynthetic systems for kinase-oriented natural product discovery. A conceptual framework summarizing the integrated discovery-to-translation pipeline for EPF-derived TKIs is presented in Fig. 2. For clarity, this review distinguishes between (i) metabolites that directly inhibit tyrosine kinases based on biochemical or structural evidence and (ii) metabolites that modulate kinase-associated signalling pathways without definitive evidence of direct kinase inhibition. Both categories are relevant to biotechnology-driven natural product discovery but differ substantially in their level of mechanistic validation. Only a small number of fungal metabolites currently satisfy the criteria for validated tyrosine kinase inhibitors, whereas considerably more compounds exhibit indirect modulation of kinase-regulated signalling pathways.

### Direct tyrosine kinase inhibition by fungal metabolites

Definitive evidence for tyrosine kinase inhibitor (TKI) activity requires direct biochemical or structure-guided validation of kinase inhibition. Although only few entomopathogenic fungi (EPF)-derived metabolites have undergone comprehensive kinase-focused evaluation, several fungal compounds exhibit measurable activity against receptor and non-receptor tyrosine kinases (Zhang et al. 2026). One notable example is the fungal metabolite (–)-TAN-2483B, which inhibits Bruton’s tyrosine kinase (BTK) based on integrated biochemical, structural, and computational analyses (McCone et al. 2024). Mechanistic studies demonstrated direct interaction with the kinase active site and provided insight into structure–activity relationships relevant to kinase binding and inhibitor optimization (Arter et al. 2022). Such studies represent important benchmarks because they integrate purified kinase assays, molecular modeling, and functional validation, thereby reducing uncertainty associated with indirect pathway interpretation. To distinguish the current level of evidence, Table 1 summarizes representative metabolites derived from entomopathogenic fungi that have demonstrated either direct tyrosine kinase inhibition or modulation of kinase-associated signaling pathways. This distinction reflects the present evidence base and should not be interpreted as indicating that all listed metabolites are validated tyrosine kinase inhibitors. Representative fungal metabolites with documented kinase or kinase-pathway activity are summarized in Table 1. Additional fungal metabolites, including cytochalasins, diketopiperazines, and several polyketides, also exhibit activity against kinase-associated signaling pathways, although mechanistic specificity varies among studies (Arter et al. 2022). Certain trichothecene-related metabolites, including roridin derivatives, inhibit fibroblast growth factor receptor (FGFR)-associated signaling, insulin-like growth factor receptor (IGF-1R), platelet-derived growth factor receptor beta (PDGFRβ), and tropomyosin receptor kinase B (TrkB) in cellular systems (Isaka et al. 1999; Li et al. 2019). In some cases, inhibition has been associated with reduced ATP binding or covalent interaction with catalytic kinase residues. However, because many trichothecenes are highly cytotoxic, distinguishing selective kinase inhibition from generalized toxicity remains challenging. The limited number of experimentally validated fungal TKIs likely reflects historical emphasis on antimicrobial, insecticidal, and general cytotoxic activities rather than kinase-focused screening. This disparity likely reflects research priorities rather than limited biosynthetic capacity. Historically, fungal natural product programs emphasized phenotypic bioactivity, whereas systematic kinase-panel screening, target-engagement studies, and phosphoproteomic profiling have only recently become accessible. Consequently, many fungal metabolite libraries, particularly those derived from EPF, remain largely unexplored using contemporary kinase-discovery platforms. Consequently, EPF metabolite libraries remain substantially underexplored using modern kinase-panel technologies, suggesting considerable untapped discovery potential. Collectively, the available studies reveal an important trend. Whereas most EPF metabolites have been evaluated through pathway-level or phenotypic assays, only a small number have undergone rigorous biochemical and structural validation of direct kinase inhibition. This imbalance reflects historical screening priorities rather than compelling evidence that EPF possess limited kinase-targeting potential.

**Table 1:** Representative metabolites derived from entomopathogenic fungi with experimentally validated tyrosine kinase inhibition or kinase-associated signaling activity

Compound	Fungal source	Chemical class	Kinase/pathway	Assay type	Reference
Beauvericin	*Beauveria bassiana*	Cyclodepsipeptide	MAPK/PI3K/Akt, apoptosis signaling	Cellular (mammalian cell lines)	Mallebrera et al. 2018;
Destruxin A	*Metarhizium anisopliae*	Cyclodepsipeptide	MAPK signaling, immune signalling pathways	Cellular (insect + mammalian models)	Dornetshuber-Fleiss et al. 2013;
Oosporein	*Beauveria bassiana*	Polyketide	Oxidative stress pathways (linked to kinase signaling)	Cellular	Fan et al. 2017
Cordycepin (3′-deoxyadenosine)	*Cordyceps militaris*	Nucleoside analogue	mTOR/AMPK signaling	Cellular + biochemical	Ashraf et al. 2020
Bassianolide	*Beauveria bassiana*	Cyclodepsipeptide	Calcium signaling, indirect kinase modulation	Cellular	Wang et al. 2021
Cyclosporin A	*Tolypocladium inflatum* (entomopathogenic lineage)	Cyclic peptide	Calcineurin pathway (phosphatase–kinase axis)	Biochemical + cellular	Yang et al. 2018

Direct tyrosine kinase inhibition refers to compounds supported by biochemical and/or structural evidence of kinase target engagement, whereas kinase-associated signaling activity denotes modulation of signaling pathways without definitive evidence of direct kinase inhibition. Only metabolites derived from entomopathogenic fungi are included

### Indirect modulation of kinase-associated signaling pathways

Beyond direct inhibition, many fungal metabolites modulate kinase-regulated signaling pathways implicated in cancer and inflammatory diseases, including MAPK, PI3K/Akt, mTOR, JAK/STAT, and NF-κB signaling (Peng et al. 2022). Although such activities do not establish definitive tyrosine kinase inhibition, they may identify compounds interacting with upstream kinase regulators or associated signaling networks. Several metabolites produced by entomopathogenic fungi (EPF) and related fungi modulate MAPK- and PI3K/Akt-associated pathways in mammalian systems (Tóthová et al. 2021). Beauvericin has been associated with apoptosis induction, oxidative stress, mitochondrial dysfunction, and suppression of Akt-related survival signaling in multiple cancer cell lines. Destruxins similarly influence calcium signaling, immune regulation, stress-associated kinase pathways, and apoptosis-related signaling events (Dornetshuber-Fleiss et al. 2013). Cordycepin from *Cordyceps* species modulates AMPK, mTOR, MAPK, and NF-κB pathways linked to inflammation, metabolic regulation, and tumor progression (Agarwal et al. 2015; Radhi et al. 2021). Importantly, kinase pathway modulation may occur through mechanisms beyond direct kinase inhibition, including oxidative stress induction, membrane perturbation, mitochondrial dysfunction, transcriptional regulation, and interference with upstream receptor activation. Consequently, pathway-level studies require cautious interpretation, particularly when conclusions rely solely on downstream signaling or transcriptional markers. Importantly, modulation of kinase-regulated signaling does not necessarily indicate direct kinase inhibition because alterations in phosphorylation networks may arise from upstream receptor activation, oxidative stress, mitochondrial dysfunction, calcium dysregulation, or broader cellular stress responses. Establishing direct kinase inhibition therefore requires complementary biochemical and structural validation beyond pathway-level observations. Nevertheless, such observations remain valuable in early stage biotechnology pipelines because they identify signaling-active metabolites suitable for mechanistic screening and biochemical validation.

Comparison of available studies further indicates that pathway modulation is considerably more common than direct kinase inhibition among currently characterized EPF metabolites. Although these observations cannot establish kinase target engagement, they provide a rational basis for prioritizing selected metabolites for subsequent biochemical kinase screening.

### Structural features associated with kinase-related activity

Several structural features recurrently observed in fungal metabolites may contribute to kinase-related bioactivity. Fungal secondary metabolites commonly possess rigid cyclic frameworks, aromatic systems, heterocyclic moieties, hydrogen bond donor–acceptor motifs, and electrophilic functionalities that facilitate interactions with ATP-binding pockets or regulatory kinase domains (Shankar and Sharma 2022). Such features are frequently observed in polyketides, diketopiperazines, cyclodepsipeptides, and hybrid PKS–NRPS metabolites produced by entomopathogenic fungi (EPF) and related fungi. Macrocyclic metabolites such as beauvericin and destruxins exhibit conformational rigidity and membrane-interacting properties associated with signaling-related protein interactions. Similarly, fungal alkaloids and hybrid metabolites often contain nitrogen-rich heterocyclic systems compatible with kinase-binding pharmacophores reported in synthetic tyrosine kinase inhibitors (TKIs) (Wang et al. 2023a, b; Heilos et al. 2017; Patra et al. 2024). Tailoring modifications including methylation, hydroxylation, halogenation, glycosylation, and epoxidation further expand structural diversity and may influence target selectivity, membrane permeability, and bioactivity. These structural features are important because they originate from modular biosynthetic systems amenable to engineering. PKS, NRPS, and hybrid assembly line enzymes provide opportunities for pathway manipulation, combinatorial biosynthesis, and rational scaffold diversification to generate metabolites with improved kinase selectivity and pharmacological properties.

### Methodological considerations and evidence quality

Interpretation of fungal kinase-related activity requires careful evaluation of experimental design and evidence quality. Earlier studies frequently relied on cytotoxicity assays, apoptosis markers, or downstream signaling readouts without direct biochemical validation of kinase inhibition. Current standards increasingly incorporate purified kinase assays, kinase selectivity profiling, molecular docking, structural biology, phosphoproteomics, and orthogonal validation strategies to establish reliable target engagement (Rüegger et al. 2025; Zhang et al. 2023a, b). For entomopathogenic fungi (EPF)-derived metabolites, this methodological gap represents both a limitation and an opportunity. The limited number of validated kinase-focused studies likely reflects insufficient screening rather than absence of activity. A further limitation is that conventional natural product screening pipelines frequently prioritize antimicrobial or cytotoxic phenotypes before target-specific characterization. While efficient for lead identification, these approaches may overlook selective kinase inhibitors lacking pronounced phenotypic activity or misclassify signaling-active metabolites as nonspecific cytotoxins. Accordingly, absence of biochemical kinase validation should not be interpreted as absence of kinase-directed potential, but rather as a reflection of the limited application of modern kinase-screening platforms to EPF metabolite libraries. Advances in high-throughput kinase panels, metabolomics-guided fractionation, AI-assisted target prediction, and multi-omics integration now support systematic evaluation of EPF metabolite libraries against therapeutically relevant kinase targets (Miduturu et al. 2011; Zhang et al. 2023a, b; Chigozie and Esimone 2026b). A major limitation of the current evidence base is the lack of standardized validation workflows across studies. Differences in experimental models, kinase panels, endpoint selection, and mechanistic validation make direct comparison between reports difficult and likely contribute to underestimation of genuinely kinase-active fungal metabolites.

Collectively, available evidence supports fungal metabolites as chemically diverse and biologically relevant resources for kinase-oriented discovery. Although a few EPF-derived compounds currently satisfy criteria for validated tyrosine kinase inhibitors (TKIs), expanding biotechnology capabilities for fungal metabolite discovery and engineering continue to strengthen their translational potential for kinase-targeted natural product development.

## Biosynthetic basis of TKI-relevant metabolites

Structural diversity of fungal secondary metabolites is driven by specialized biosynthetic gene clusters (BGCs) encoding pathways for metabolite assembly, modification, transport, and regulation. Increasing genomic evidence indicates that entomopathogenic fungi (EPF) possess a large number and largely underexplored biosynthetic capacities capable of producing chemically diverse metabolites with potential kinase-related activities (Bihal et al. 2023; Pimkhonburee et al. 2026). Consequently, understanding the regulation and engineering potential of these biosynthetic systems is central to biotechnology-driven discovery of tyrosine kinase inhibitor (TKI)-relevant fungal metabolites.

### Biosynthetic gene cluster diversity in entomopathogenic fungi

Whole-genome sequencing has revealed a marked disparity between the large number of predicted biosynthetic gene clusters (BGCs) in entomopathogenic fungi (EPF) and the comparatively limited number of experimentally characterized metabolites. This occurs because most BGCs are cryptic or silent—meaning their genes remain turned off under standard laboratory culture conditions (Zhang et al. 2020). Genomes of *Beauveria bassiana*, *Metarhizium robertsii*, *Cordyceps militaris*, and related fungi encode a number polyketide synthase (PKS), non-ribosomal peptide synthetase (NRPS), terpene synthase, and hybrid PKS–NRPS clusters, many of which remain transcriptionally silent under standard cultivation conditions (Singh et al. 2017; Lim et al. 2012). This hidden biosynthetic capacity highlights EPF as reservoirs of cryptic metabolic diversity with potential relevance to kinase-oriented natural product discovery.

Bioinformatic platforms including antiSMASH, PRISM, SMURF, and AI-assisted genome mining pipelines now permit systematic identification and annotation of fungal BGCs (Blin et al. 2024; Khaldi et al. 2010). These tools support prediction of core biosynthetic enzymes, tailoring domains, precursor specificity, and putative metabolite classes directly from genomic data. Integration of machine learning with genomic, transcriptomic, metabolomic, and phylogenetic datasets is further improving prioritization of cryptic clusters and facilitating targeted pathway activation. Several characterized EPF metabolites associated with signaling-related activities originate from modular biosynthetic pathways. Beauvericin and bassianolide are synthesized by NRPS systems, whereas oosporein and tenellin derive from PKS-associated pathways. Hybrid PKS–NRPS systems are particularly important because they generate structurally diverse scaffolds combining peptide and polyketide biosynthetic features (Lin et al. 2026; Iacovelli et al. 2022), thereby increasing the likelihood of kinase-interacting pharmacophores. Although genome mining has dramatically expanded the catalogue of predicted fungal biosynthetic pathways, experimental characterization has progressed considerably more slowly. Consequently, current understanding of EPF metabolic diversity remains constrained more by functional validation than by genome availability.

### Biosynthetic logic underlying kinase-relevant scaffolds

**Fig. 3:**
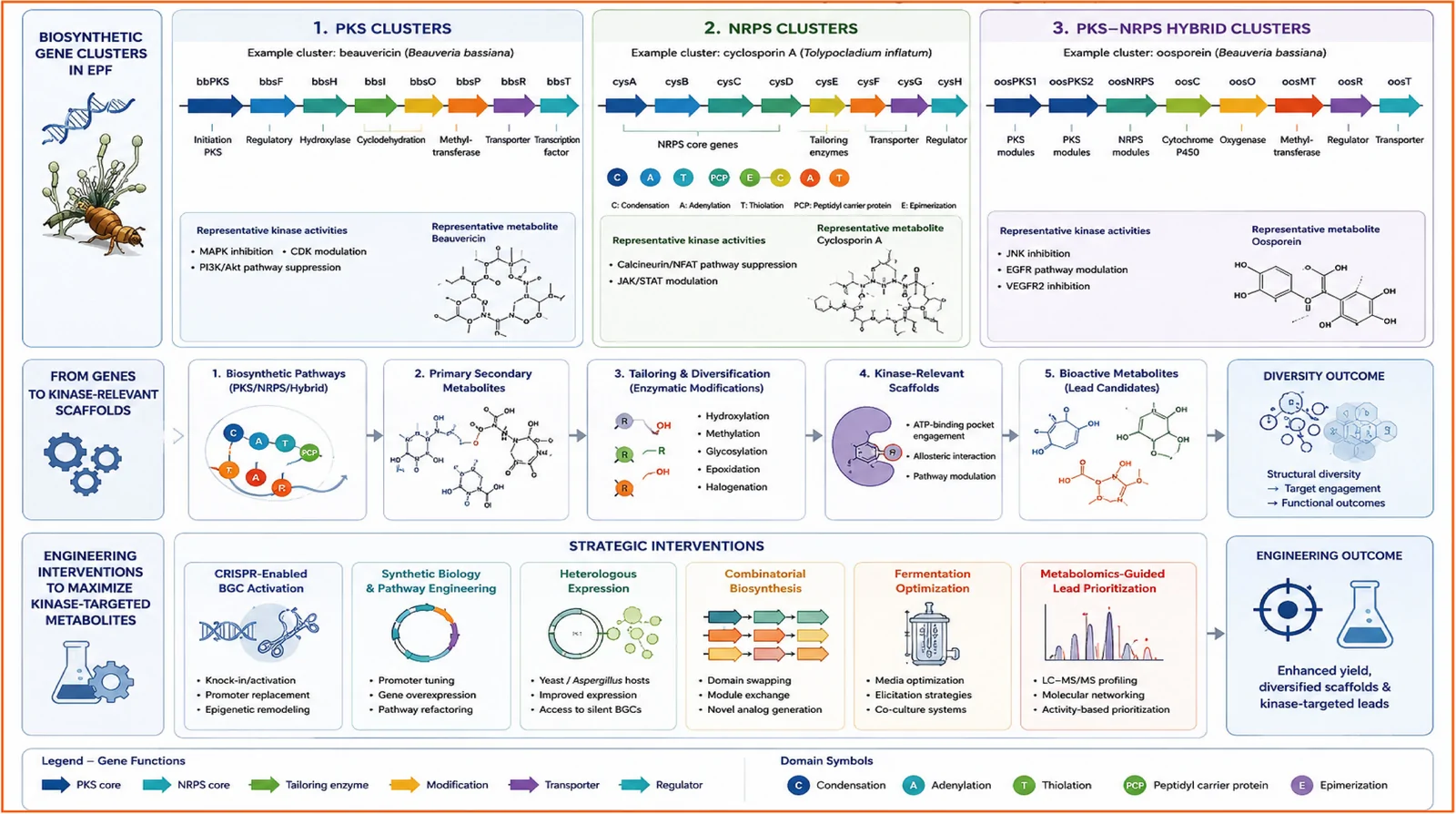
Biosynthetic Gene Cluster (BGC) Architectures linked to kinase-relevant secondary metabolites—Schematic representation of canonical and hybrid BGC frameworks (PKS, NRPS, and PKS–NRPS) highlighting modular domain organization, assembly line logic, and key tailoring enzymes (e.g., methyltransferase, Glycosylase, Oxygenase) that drive structural diversification. The figure maps precursor selection, chain elongation, cyclization, and post-assembly modifications to the generation of chemically complex scaffolds with kinase-inhibitory potential, while indicating regulatory elements and engineering intervention points (promoter refactoring, domain swapping, pathway activation) for enhanced discovery and production (authors concept image)

Fungal secondary metabolite biosynthesis follows modular assembly line logic in which biosynthetic enzymes iteratively extend, cyclize, and modify precursor molecules. Polyketide synthases (PKSs) generate polyketide scaffolds from acetate-derived units, whereas non-ribosomal peptide synthetases (NRPSs) assemble peptide-based metabolites from amino acid substrates (Fig. 3). Hybrid PKS–NRPS systems further expand structural and stereochemical diversity. These biosynthetic systems generate molecular features frequently associated with kinase-binding interactions, including aromatic rings, macrocycles, heterocycles, hydrogen bond donor–acceptor motifs, and electrophilic groups. Tailoring enzymes such as methyltransferases, cytochrome P450 monooxygenases, glycosyltransferases, halogenases, epoxidases, and oxidoreductases further diversify these scaffolds through post-assembly modifications that may influence target affinity, selectivity, membrane permeability, and bioactivity. From a biotechnology perspective, modular biosynthetic architecture provides substantial opportunities for pathway engineering. Domain swapping, module recombination, precursor-directed biosynthesis, and rational refactoring of PKS and NRPS systems can generate structural analogues with altered pharmacological properties (Butler et al. 2026; Bozhüyük et al. 2024). Advances in synthetic biology have also enabled redesign of fungal biosynthetic pathways in heterologous hosts, thereby improving metabolite accessibility and scaffold diversification.

### Regulation of secondary metabolism in entomopathogenic fungi

Secondary metabolite production in entomopathogenic fungi (EPF) is tightly regulated by environmental, nutritional, developmental, and stress-associated signals. A large number of biosynthetic gene clusters (BGCs) remain silent because their expression depends on complex transcriptional and epigenetic networks that are not activated under routine laboratory conditions (Zhang et al. 2020). Consequently, understanding these regulatory mechanisms is essential for accessing cryptic kinase-relevant metabolites. Global regulators including LaeA, VeA, VelB, and other velvet complex components play central roles in coordinating fungal development and secondary metabolism (Chen et al. 2026; Bayram et al. 2008). These regulators influence chromatin accessibility, transcriptional activation, and environmental responsiveness of multiple BGCs. Histone modification, DNA methylation, and chromatin remodeling also contribute to pathway silencing and activation. Accordingly, epigenetic modulation using histone deacetylase or DNA methyltransferase inhibitors has emerged as an effective strategy for activating cryptic metabolite production in filamentous fungi (VanderMolen et al. 2014). Environmental mimicry strategies are similarly important for activating silent pathways in EPF. Cultivation under insect-mimicking or stress-induced conditions can stimulate otherwise silent BGCs. Oxidative stress, nutrient limitation, co-culture interactions, osmotic stress, pH variation, and host-derived signals have all been associated with altered secondary metabolism. The One Strain–Many Compounds (OSMAC) strategy exploits these responses by systematically modifying cultivation parameters to maximize chemical diversity (Zheng et al. 2025). Advances in transcriptomics, metabolomics, and systems biology now permit more precise interrogation of these regulatory networks. Integration of multi-omics datasets can identify co-expressed genes, pathway-specific regulators, and environmental triggers associated with metabolite production (Wörheide et al. 2021). Such approaches are increasingly important for linking cryptic BGCs to bioactive metabolites with potential kinase-related activities.

### Engineering opportunities for kinase-oriented metabolite discovery

Increasing genetic tractability of entomopathogenic fungi (EPF) and related filamentous fungi has expanded opportunities for biotechnology-driven pathway engineering. CRISPR/Cas-based genome editing now permits targeted activation, deletion, and refactoring of fungal biosynthetic gene clusters (BGCs) with increasing precision (Wang et al. 2023a, b). Promoter replacement and transcription factor overexpression can further bypass native regulatory constraints and activate otherwise silent pathways (Zhang et al. 2017). Heterologous expression in genetically tractable hosts such as *Aspergillus nidulans*, *Aspergillus oryzae*, and *Saccharomyces cerevisiae* enables improved pathway characterization and scalable metabolite production. Coupling biosynthetic engineering with kinase-focused screening platforms may accelerate discovery of kinase-active fungal metabolites while improving production feasibility. Collectively, integration of genome mining, synthetic biology, metabolomics-guided prioritization, and high-throughput kinase assays is transforming EPF from underexplored natural product sources into programmable microbial platforms for kinase-oriented therapeutic discovery.

## Biotechnological production strategies

Efficient translation of entomopathogenic fungi (EPF)-derived kinase-active metabolites requires scalable and reproducible production systems. However, many fungal secondary metabolites are produced at low yields or originate from poorly expressed cryptic biosynthetic pathways. Consequently, advances in fermentation engineering, metabolic engineering, and synthetic biology are increasingly being applied to optimize metabolite production and establish EPF as tractable microbial platforms for kinase-oriented natural product discovery.

### Native strain optimization and cultivation strategies

Optimization of native strains remains a key approach for improving fungal secondary metabolite production. In entomopathogenic fungi (EPF), biosynthesis is highly influenced by nutritional and environmental conditions, including carbon and nitrogen sources, pH, aeration, temperature, osmotic stress, trace elements, and cultivation time (Mascarin et al. 2024). Modulation of these parameters can significantly affect metabolite yield and chemical diversity. Media engineering strategies are widely used to redirect biosynthetic flux through nutrient manipulation and controlled metabolic stress. Carbon-to-nitrogen balance, phosphate limitation, and precursor supplementation may enhance production of specific metabolite classes, including non-ribosomal peptides and polyketides. The One Strain–Many Compounds (OSMAC) approach further exploits fungal metabolic plasticity by altering cultivation conditions to activate silent or weakly expressed biosynthetic pathways (de Andrade et al. 2025; Zheng et al. 2025). Variations in salinity, oxygen availability, co-cultivation, and stress exposure can induce metabolites absent under standard conditions. In EPF, host-mimicking environments may be particularly effective because many biosynthetic pathways evolved under insect-associated selective pressures. Chemical and biological elicitation strategies are also increasingly employed to activate cryptic biosynthetic gene clusters (BGCs) (Tyurin et al. 2018). Epigenetic modifiers, including histone deacetylase and DNA methyltransferase inhibitors, may relieve chromatin-mediated silencing, whereas co-culture systems can stimulate interspecies signaling linked to secondary metabolism (Asamizu 2025). These approaches are especially relevant in EPF, where many predicted BGCs remain transcriptionally inactive during conventional monoculture cultivation.

### Metabolic engineering and synthetic biology approaches

Although cultivation optimization can improve metabolite yields, native regulatory constraints often limit the scalability and reproducibility of fungal secondary metabolism. Consequently, metabolic engineering and synthetic biology provide more precise strategies for controlling biosynthetic pathway activity and improving production performance. CRISPR/Cas-based genome editing has become an important tool for fungal pathway engineering. In entomopathogenic fungi (EPF) and related filamentous fungi, CRISPR systems have enabled deletion of competing pathways, activation of silent biosynthetic gene clusters (BGCs), insertion of regulatory elements, and modification of biosynthetic enzymes (Ma et al. 2025). Promoter replacement strategies are particularly effective because many fungal BGCs remain poorly expressed under native regulation. Substitution with constitutive or inducible promoters can substantially enhance transcriptional activity and metabolite production (Shil et al. 2026). Pathway refactoring further extends these capabilities by reorganizing biosynthetic pathways into genetically optimized modules (Li et al. 2021). Approaches include removal of native regulatory elements, modular reconstruction of PKS and NRPS systems, codon optimization, and redistribution of biosynthetic genes within heterologous hosts (Li et al. 2021). Such strategies improve pathway control and facilitate combinatorial biosynthesis for generation of structurally diverse analogues. Synthetic biology also enables optimization of precursor supply pathways and intracellular metabolic flux. Because fungal secondary metabolism competes with primary metabolism for precursors such as acetyl-CoA, malonyl-CoA, amino acids, and NADPH, engineering central metabolic pathways can significantly improve metabolite productivity (Ghaempanah et al. 2026; Liu et al. 2025; Chen et al. 2024). Systems biology and genome-scale metabolic modelling are increasingly applied to identify metabolic bottlenecks and optimize biosynthetic efficiency. Importantly, the modular organization of fungal biosynthetic systems provides substantial opportunities for kinase-oriented scaffold engineering. Combinatorial biosynthesis involving PKS, NRPS, and tailoring enzyme modifications may expand structural diversity and generate analogues with improved selectivity, potency, and pharmacokinetic properties.

### Heterologous expression platforms

Heterologous expression has become a central strategy for functional characterization and scalable production of fungal secondary metabolites. Many entomopathogenic fungi (EPF) are genetically less tractable, slow growing, or difficult to cultivate at industrial scale, making direct metabolite production challenging (Jia et al. 2024). Expression of fungal biosynthetic gene clusters (BGCs) in well-characterized microbial hosts can therefore improve pathway accessibility, regulatory control, and production consistency. Filamentous fungi such as *Aspergillus nidulans* and *Aspergillus oryzae* are among the most widely used heterologous hosts because they possess eukaryotic post-translational machinery compatible with fungal biosynthetic enzymes (Sheng et al. 2025; Yuan et al. 2022). Recent work has further demonstrated that automated heterologous expression in *A. oryzae* enables efficient exploration of cryptic fungal biosynthetic gene clusters and substantially accelerates natural product discovery (Yuan et al. 2022). These systems support expression of large PKS and NRPS pathways, efficient precursor utilization, and appropriate subcellular compartmentalization. Their established genetic toolkits also facilitate promoter engineering, pathway integration, and regulatory optimization (Sheng et al. 2025). Yeast systems, particularly *Saccharomyces cerevisiae*, are increasingly used for fungal pathway reconstruction because of their rapid growth, genetic tractability, and compatibility with synthetic biology workflows. Yeast platforms are especially valuable for modular pathway assembly, combinatorial engineering, and high-throughput screening. However, limitations related to codon usage, precursor availability, protein folding, and expression of large multidomain enzymes may reduce productivity in some cases. Despite these advantages, heterologous systems present important technical challenges. Expression of large fungal BGCs often requires coordinated regulation of multiple genes, specialized cofactors, transporter proteins, and pathway-specific regulatory elements (Zhgun 2023). In addition, some fungal metabolites may exhibit host toxicity or depend on subcellular environments not fully replicated in heterologous hosts. Consequently, selection and optimization of suitable expression platforms remain highly pathway dependent. These observations suggest that heterologous expression should be viewed as a complementary rather than universally superior production strategy. Selection of an appropriate production host remains dependent on pathway architecture, enzyme compatibility, and the physicochemical characteristics of the target metabolite.

### Fermentation and downstream processing

Efficient fermentation and downstream processing are essential for scalable production of fungal secondary metabolites. Both submerged fermentation (SmF) and solid-state fermentation (SSF) are widely used for cultivation of entomopathogenic fungi (EPF), each offering distinct advantages and limitations. SmF provides superior control of pH, aeration, nutrient delivery, agitation, and process monitoring, making it well suited for industrial scale-up and reproducible metabolite production. However, some EPF metabolites are preferentially produced under surface-associated or stress-induced conditions that are not fully replicated in liquid cultures. SSF more closely mimics the natural ecological environment of filamentous fungi and may stimulate expression of otherwise poorly expressed biosynthetic pathways. SSF has been associated with enhanced sporulation and increased secondary metabolite production, although large-scale implementation remains constrained by limitations in heat transfer, oxygen diffusion, and process standardization (Bamidele et al. 2025). Downstream processing remains a major bottleneck in fungal metabolite commercialization. Low metabolite yields, compound instability, and co-production of structurally related metabolites can complicate purification workflows (Majid, 2025). Conventional solvent extraction and chromatographic methods remain widely used, although metabolomics-guided fractionation, automated purification systems, membrane technologies, and in situ product recovery strategies are increasingly improving process efficiency and scalability. Collectively, advances in fermentation optimization, synthetic biology, heterologous expression, and bioprocess engineering are strengthening the feasibility of exploiting EPF as scalable microbial platforms for kinase-oriented natural product production (Mou & Hai 2026). However, successful translation will depend on overcoming challenges related to reproducibility, selectivity, toxicity, regulatory approval, and industrial scalability.

## Translational challenges

Despite increasing interest in EPF-derived kinase-relevant metabolites, translation remains constrained because very few compounds have progressed beyond pathway-level observations to validated kinase-specific mechanisms.

### Selectivity and mechanistic specificity

A major translational limitation of fungal kinase-active metabolites is insufficient target selectivity. Because protein kinases possess highly conserved ATP-binding domains, selective inhibition remains challenging, increasing the risk of off-target effects and nonspecific cytotoxicity. Many entomopathogenic fungi (EPF)-derived metabolites associated with kinase-related activity have been evaluated primarily using cellular phenotypes or downstream signaling markers rather than comprehensive kinase profiling (Pimkhonburee et al. 2026). Consequently, distinguishing direct kinase inhibition from indirect pathway perturbation remains difficult. Metabolites that induce oxidative stress, membrane disruption, mitochondrial dysfunction, or transcriptional dysregulation may alter phosphorylation networks without directly targeting tyrosine kinases. This limitation is particularly relevant for structurally reactive metabolites such as trichothecenes and macrocyclic toxins, which may exhibit potent antiproliferative activity but limited therapeutic selectivity. Although such compounds may provide valuable lead scaffolds, substantial medicinal chemistry optimization and mechanistic validation are often required. Modern translational workflows increasingly integrate kinase-panel assays, phosphoproteomics, structural biology, and orthogonal genetic validation to establish reliable target engagement (Adedokun et al. 2026). Incorporation of these approaches into fungal metabolite discovery pipelines will be essential for improving translational relevance and reducing late-stage attrition.

### Toxicity and therapeutic window limitations

Toxicity remains a major challenge in development of fungal natural products. Many secondary metabolites produced by entomopathogenic fungi (EPF) evolved as defense or virulence-associated compounds and therefore exhibit potent cytotoxic or immunomodulatory activities (Pimkhonburee et al. 2026). Although these properties may contribute to therapeutic activity, they can also result in narrow therapeutic windows and poor safety profiles. Several fungal metabolites associated with kinase-related pathways exhibit dose-dependent toxicity in mammalian systems. In some cases, cytotoxicity occurs independently of kinase inhibition, limiting translational potential despite apparent pathway activity (Mo et al. 2023). In addition, unfavourable physicochemical properties, including poor solubility, metabolic instability, limited bioavailability, and suboptimal tissue distribution, may further restrict therapeutic applicability. Addressing these limitations will require integration of biosynthetic engineering, medicinal chemistry, structure-guided optimization, and targeted delivery strategies (Ligarda-Samanez et al. 2025). Engineering less-toxic analogues through pathway modification or tailoring enzyme manipulation may improve safety while preserving biological activity. Similarly, advanced formulation and nanotechnology-based delivery systems may enhance selectivity and reduce systemic toxicity.

### Scalability and production reproducibility

Scalable production remains a major barrier to commercialization of fungal secondary metabolites. Many entomopathogenic fungi (EPF)-derived compounds are produced at low yields, exhibit unstable expression patterns, or depend on tightly regulated environmental conditions that are difficult to reproduce during industrial cultivation (Bhattacharyya et al. 2026). In addition, strain degeneration during repeated subculturing may reduce sporulation efficiency, alter regulatory responses, and decrease metabolite productivity, thereby compromising process stability and industrial feasibility. Cryptic biosynthetic pathways further contribute to production variability. Many kinase-relevant metabolites originate from silent or weakly expressed biosynthetic gene clusters (BGCs) whose activation depends on specific stress or ecological conditions that are difficult to replicate at scale (Zhang et al. 2019). Variations in oxygen transfer, nutrient gradients, shear stress, pH, and fermentation geometry may substantially influence metabolite output. Advances in metabolic engineering, synthetic biology, and bioprocess optimization are increasingly addressing these limitations. CRISPR-based pathway activation, promoter refactoring, heterologous expression, and systems biology–guided optimization may improve production stability and scalability. Nevertheless, industrial translation will require robust fermentation workflows capable of maintaining reproducible metabolite yields under economically viable conditions (Sokra et al. 2026).

### Regulatory and intellectual property considerations

Regulatory approval of fungal-derived therapeutics remains challenging because fungal metabolites require extensive evaluation of purity, toxicity, batch consistency, manufacturing reproducibility, and contaminant profiles. Concerns regarding mycotoxin-associated toxicity may further complicate regulatory acceptance, particularly for metabolites structurally related to known fungal toxins. Standardization also represents a major translational barrier. Variability in strain genetics, cultivation conditions, extraction procedures, and downstream processing may substantially influence metabolite composition and biological activity. Consequently, reproducible manufacturing workflows and validated quality-control frameworks are essential for regulatory approval. Intellectual property (IP) considerations may further affect translational feasibility because naturally occurring fungal metabolites often present patentability limitations (Kantaros et al. 2025). Biotechnology-driven diversification strategies, including combinatorial biosynthesis, pathway engineering, and synthetic biology–enabled analogue generation, may therefore strengthen commercial value and IP protection.

### Integrating biotechnology with translational development

Overcoming translational barriers will require integration of fungal biotechnology, systems biology, pharmacology, medicinal chemistry, and industrial bioprocess engineering. Future progress will depend not only on discovery of kinase-active metabolites, but also on development of scalable and mechanistically validated production platforms (Chigozie and Esimone 2026b). Advances in AI-assisted target prediction, multi-omics integration, genome-scale metabolic modelling, and high-throughput screening are increasingly improving fungal natural product discovery pipelines. Integration of these technologies with engineered fungal production systems may enhance metabolite prioritization, scalability, and translational feasibility. Despite remaining challenges, ongoing advances in microbial biotechnology continue to strengthen the feasibility of exploiting entomopathogenic fungi (EPF) as sustainable and engineerable platforms for kinase-oriented therapeutic discovery.

## Future directions

Advances in fungal genomics, synthetic biology, metabolomics, and computational biotechnology are expanding opportunities for systematic exploitation of entomopathogenic fungi (EPF) in kinase-oriented natural product discovery. Although validated tyrosine kinase inhibitor (TKI) activity remains limited for many EPF-derived metabolites, emerging technologies are improving identification, engineering, and translational development of fungal secondary metabolites. Consequently, future research should prioritize rigorous biochemical validation alongside continued discovery of novel fungal metabolites with kinase-oriented potential. Future progress will depend on integration of genome-guided discovery, mechanism-driven screening, and scalable biotechnological production platforms.

### AI-assisted genome mining and pathway prioritization

Artificial intelligence (AI) and machine learning are increasingly advancing biosynthetic gene cluster (BGC) discovery and metabolite prediction in fungal biotechnology. Although conventional genome mining tools have improved identification of PKS, NRPS, terpene, and hybrid pathways, prioritization of biologically relevant cryptic clusters remains challenging because fungal genomes often encode far more BGCs than experimentally characterized metabolites (Zhang et al. 2025a, b). AI-assisted platforms integrating genomic, transcriptomic, metabolomic, and phylogenetic datasets are improving prediction of kinase-relevant pathways, substrate specificity, tailoring enzyme function, and potential bioactivity directly from biosynthetic sequence information (Yetgin 2025). Such approaches may accelerate identification of fungal metabolites possessing kinase-interacting scaffolds before labor-intensive isolation and characterization. These technologies are particularly relevant for entomopathogenic fungi (EPF), where many biosynthetic pathways remain underrepresented in current natural product databases. Integration of AI-guided prioritization with experimental validation workflows may therefore improve discovery efficiency while reducing rediscovery of known metabolites.

### Coupling metabolomics with kinase-focused screening

Integration of metabolomics-driven profiling with high-throughput kinase screening may substantially improve fungal natural product discovery. Traditional fungal screening approaches have relied heavily on phenotypic bioassays or general cytotoxicity assays, which often lack mechanistic specificity and may overlook selective kinase-active metabolites. Advances in LC–MS/MS metabolomics, molecular networking, imaging mass spectrometry, and feature-based molecular annotation now permit more comprehensive characterization of fungal metabolomes. Integration of these approaches with kinase-panel assays, phosphoproteomics, and pathway-specific reporter systems may improve identification of metabolites targeting therapeutically relevant signaling networks (David et al. 2025). Multi-omics integration may further facilitate linkage between cryptic biosynthetic gene clusters (BGCs) and bioactive metabolites. Simultaneous analysis of transcriptional activation, metabolite production, and kinase-related bioactivity under varying environmental conditions could improve mapping of genotype–metabolite–bioactivity relationships in entomopathogenic fungi (EPF) (Fan et al. 2025). Such workflows are increasingly consistent with data-driven microbial biotechnology approaches for natural product discovery. Importantly, future progress will depend not simply on generating larger metabolite libraries but on integrating discovery platforms with mechanism-driven validation workflows capable of distinguishing direct kinase inhibitors from broader signalling modulators at an early stage of development.

### Engineering EPF-derived scaffolds for translational optimization

Although several fungal metabolites exhibit promising kinase-related activities, only few possess physicochemical or pharmacological properties suitable for direct therapeutic application. Consequently, future efforts will likely focus not only on discovery of novel metabolites, but also on engineering existing fungal scaffolds into more drug-like molecules. Synthetic biology and combinatorial biosynthesis provide important opportunities for structural diversification of fungal metabolites (Chigozie and Esimone 2026b). Manipulation of PKS, NRPS, and tailoring enzyme domains may generate analogues with improved selectivity, reduced toxicity, and enhanced pharmacokinetic properties (Jayachandran et al. 2026). Advances in pathway refactoring and modular biosynthetic engineering are increasingly enabling genetic redesign of fungal metabolites beyond conventional medicinal chemistry approaches. Coupling biosynthetic engineering with structure-guided computational modelling may further facilitate optimization of kinase-interacting metabolites (Skellam et al. 2024). AI-assisted molecular docking, molecular dynamics simulations, and predictive pharmacology platforms may improve prioritization of engineered analogues before large-scale production and biological evaluation.

### Expanding heterologous and cell-factory platforms

Future scalability of entomopathogenic fungi (EPF)-derived metabolites will depend heavily on development of robust microbial production platforms. Native EPF often exhibit slow growth, strain instability, and tightly regulated secondary metabolism, limiting industrial feasibility. Consequently, heterologous expression systems remain central to translational development. Emerging fungal and yeast chassis optimized for secondary metabolite production may improve pathway accessibility and yield stability (Sheng et al. 2025). Advances in genome editing, synthetic chromosomes, dynamic promoter systems, and automated strain engineering are enabling increasingly sophisticated fungal cell factories capable of expressing large and complex biosynthetic gene clusters (BGCs) (Mózsik et al. 2022; Sadravi et al. 2026). Integration of adaptive laboratory evolution, systems metabolic engineering, and bioprocess optimization may further improve industrial scalability. Cell-free biosynthetic systems and enzyme reconstitution platforms may also provide complementary strategies for production and characterization of kinase-active fungal metabolites, particularly those associated with tightly regulated or toxic biosynthetic pathways.

### EPF as sustainable platforms for kinase-oriented discovery

**Fig. 4:**
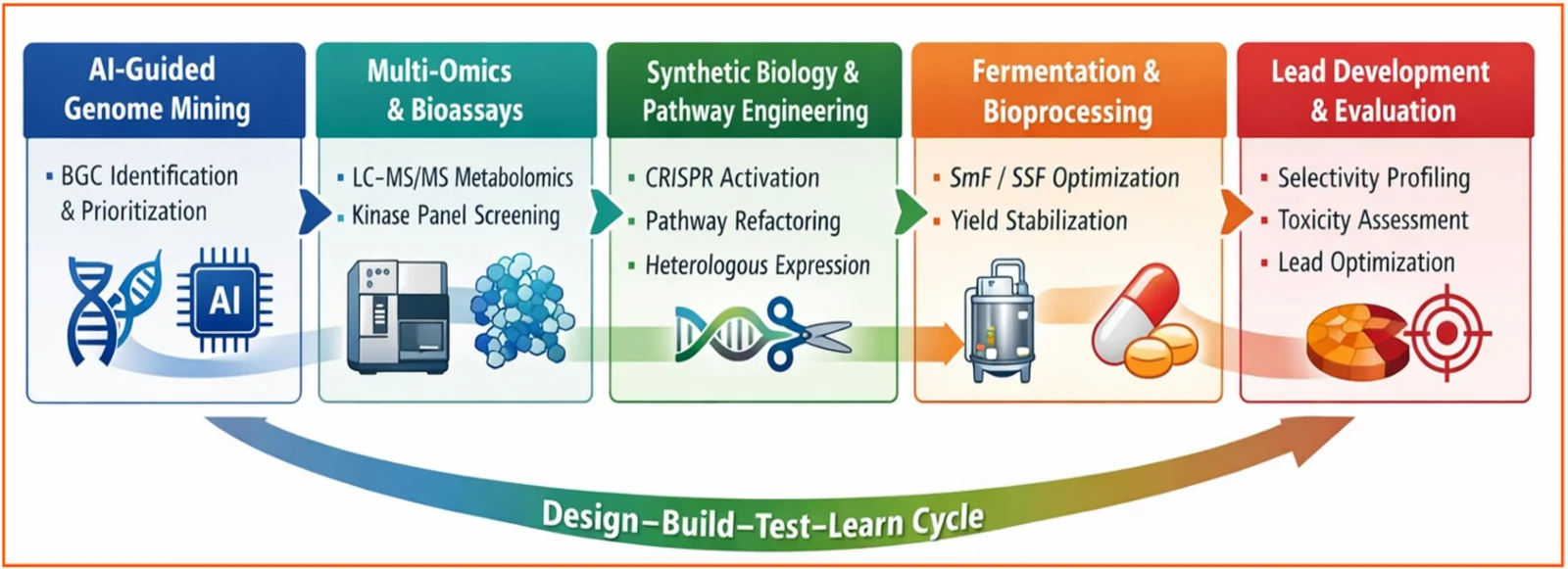
Pipeline for EPF-derived TKI discovery, from genome mining to lead optimization. Integrated biotechnological pipeline for EPF-derived tyrosine kinase inhibitor discovery, spanning AI-guided genome mining, multi-omics validation, synthetic biology–driven pathway engineering, scalable bioprocessing, and lead optimization within an iterative design–build–test–learn framework. (Authors concept image)

Limitations of conventional synthetic libraries, including restricted scaffold diversity and high attrition rates, have renewed interest in natural product–based discovery platforms. In this context, entomopathogenic fungi (EPF) represent promising microbial resources because their metabolites arise from prolonged interactions with eukaryotic hosts, increasing the likelihood of biologically relevant target engagement (Bihal et al. 2023). EPF biosynthetic systems generate structurally complex and stereochemically diverse metabolites through modular enzymatic pathways that are difficult to replicate synthetically. Such diversity may facilitate identification of novel kinase-interacting scaffolds with distinct pharmacological properties. Advances in genome mining, synthetic biology, pathway engineering, and fermentation optimization (Fig. 4) are increasingly enabling scalable production of these metabolites through sustainable microbial processes (Gong et al. 2024). Consequently, EPF are emerging not only as sources of bioactive natural products, but also as engineerable microbial platforms for kinase-oriented discovery. Continued integration of AI-assisted genome mining, metabolomics-guided screening, heterologous expression, and bioprocess engineering is likely to further enhance their translational relevance.

## Conclusions

Entomopathogenic fungi represent underexplored microbial platforms for discovering and engineering kinase-relevant secondary metabolites rather than established sources of validated tyrosine kinase inhibitors. The principal limitation of the current evidence base is not an absence of promising fungal chemistry but the relatively limited application of rigorous kinase-specific validation workflows to EPF-derived metabolites. Advances in fungal genomics, metabolomics, and synthetic biology have revealed a great number of cryptic biosynthetic capacities capable of generating structurally diverse and biologically active metabolites. Although only a limited number of EPF-derived metabolites have been experimentally validated as direct tyrosine kinase inhibitors, increasing evidence demonstrates that many modulate kinase-associated signalling pathways and therefore constitute valuable starting points for kinase-oriented natural product discovery. Accordingly, the metabolites summarized in this review should be viewed as representing different stages of mechanistic validation, ranging from pathway-level modulation to experimentally confirmed kinase inhibition. Beyond metabolite discovery, EPF are emerging as engineerable microbial platforms for biotechnology-driven production and diversification of kinase-active compounds (Chigozie and Esimone 2026b). Genome mining, CRISPR-based pathway activation, heterologous expression, metabolomics-guided screening, and synthetic biology are increasingly transforming fungal secondary metabolism into a programmable and translationally relevant biotechnology field. Nevertheless, important challenges remain, including limitations related to target selectivity, mechanistic validation, toxicity, production reproducibility, scalability, and regulatory acceptance. Addressing these challenges will require integrated approaches combining biosynthetic engineering, medicinal chemistry, nanotechnology-based delivery systems, structure-guided optimization, and early consideration of pharmacokinetic and translational barriers (Chigozie et al. 2026). Future progress will likely depend on AI-assisted genome mining, pathway refactoring, combinatorial biosynthesis, and scalable fungal cell-factory development to improve discovery efficiency and translational feasibility. Overall, current evidence supports cautious optimism. EPF should not yet be regarded as established sources of tyrosine kinase inhibitors; rather, they represent biologically plausible and biotechnologically tractable microbial platforms whose therapeutic potential remains incompletely validated and warrants systematic investigation using modern kinase-discovery technologies. Collectively, current evidence supports the growing potential of EPF as sustainable and engineerable platforms for kinase-oriented therapeutic discovery.

## Data Availability

No datasets were generated or analysed during the current study.
